# Effect of NaOCl and EDTA irrigating solutions on the cyclic fatigue resistance of EdgeTaper Platinum instruments

**DOI:** 10.1186/s12903-022-02215-0

**Published:** 2022-05-22

**Authors:** Hussam Alfawaz, Abdullah Alqedairi, Maha Alhamdan, Najla Alkhzim, Shatha Alfarraj, Ahmed Jamleh

**Affiliations:** 1 Department of Restorative Dental Sciences, College of Dentistry, King Saud University, P.O. Box 60169, Riyadh, 11545 Saudi Arabia; 2 Department of Restorative and Prosthetic Sciences, College of Dentistry, King Saud Bin Abdulaziz University for Health Sciences, Riyadh, Saudi Arabia; 3 King Abdullah International Medical Research Center, Ministry of National Guard-Health Affairs, Riyadh, Saudi Arabia

**Keywords:** Cyclic fatigue resistance, EdgeTaper Platinum, EDTA, Heat-treated instrument, ProTaper Gold, Sodium hypochlorite

## Abstract

**Background:**

To compare the solution on the cyclic fatigue resistance of EdgeTaper Platinum (ETP) instruments with that of ProTaper Gold (PTG) in the presence of ethylenediaminetetraacetic acid (EDTA) or sodium hypochlorite (NaOCl) irrigating solutions.

**Methods:**

Sixty PTG and 60 ETP instruments with the same size (#25) and taper (8%) were tested under 17% EDTA, 5.25% NaOCl, or distilled water (n = 20 each). Cyclic fatigue was tested using artificial canals that were milled in stainless steel blocks using a laser micromachining technique. The canals had a curvature angle of 60 and a curvature radius of 5 mm. The center of maximum curvature was set at 5 mm from the instrument tip. The block of artificial canals was stabilized inside a container that was filled with one of the tested solutions. The temperature was fixed at 37 °C with a tolerance limit of 1 °C. The number of cycles to fracture (NCF) was calculated and the fractured surfaces were examined using a scanning electron microscope. All statistical analyses were performed using SPSS software Version 20 (IBM-SPSS Inc., Chicago, IL) at a confidence level of 95%.

**Results:**

ETP showed higher NCF than PTG in any of the tested solutions (*P* < 0.05). Within each group, NaOCl drastically decreased the NCF compared to water and EDTA (*P* < 0.05) and changing the solution from distilled water to EDTA did not affect the fatigue resistance (*P* > 0.05).

**Conclusions:**

ETP showed improved cyclic fatigue performance compared to PTG in all tested irrigating solutions. EDTA can be used in combination with NiTi instruments during canal instrumentation without compromising the cyclic fatigue resistance of PTG and ETP instruments. However, NaOCl drastically decreased the NCF.

## Background

Endodontics has grown significantly over the past years with the significant development in manufacturing nickel–titanium (NiTi) instruments which led to improved root canal treatment outcomes [1]. These instruments have gained wide popularity ever since due to their superior characteristics derived from inherently super-elastic with excellent shape memory [2]. These properties enable the rotary NiTi instruments to prepare curved canals quicker with fewer procedural errors [3]. However, the risk of instrument separation is still a concern among clinicians which is considered as the most common procedural mishap [4]. There are two identified reasons for instrument separation: torsional overload and cyclic fatigue [5]. Cyclic fatigue develops when a NiTi file undergoes tensile-compressive stresses as a result of repetitive motions during the root canal treatment [5]. Torsional overload may occur from apically directed pressure and wedging the files’ tip into the canal overzealously [5].

The irrigating solution is used in endodontics to serve a variety of chemical, biological, and mechanical functions which are crucial for effective root canal treatment [6]. Canal instrumentation should always be carried out under copious irrigation as it is never advisable to instrument a dry canal [7]. However, the irrigating solution comes in contact with NiTi instruments which might induce instrument corrosion and deformation accelerating instrument's fatigue failure [8, 9]. Previous studies reported that irrigating solutions influence the performance of endodontic files [10–19]. The most commonly used irrigating solutions are sodium hypochlorite (NaOCl) and ethylenediaminetetraacetic acid (EDTA).

Thermal treatment is a technology used to make the phase transformation temperature close to the intracanal temperature with a high austenite finish temperature approaching 50 °C [20]. The high transformation temperature indicates that the thermally treated instrument will have some degree of martensite at a simulated intracanal temperature, and thus, greater the martensitic behavior with improved resistance to cyclic fatigue [21], flexibility [22], and fatigue crack growth resistance [23]. ProTaper Gold (PTG; Dentsply Sirona, Ballaigues, Switzerland) and EdgeTaper Platinum (ETP; EdgeEndo, Albuquerque, NM, USA) are NiTi instruments which have been manufactured with thermal treatment technology [24]. Both have the same design with a convex triangular cross-section. A previous study concluded that ETP had excellent resistance to cyclic fatigue compared with PTG when tested in distilled water [24].

In clinical practice, the instrument is often used while the canal is flooded with irrigating solutions that could have an effect on the instrument performance as shown previously on other instruments systems [10–19]. Because no data are available on the effect of irrigating solutions on ETP instrument’s cyclic fatigue resistance, the current study was undertaken to investigate the cyclic fatigue resistance of ETP file instrument in the presence of EDTA, NaOCl, or distilled water and compare it with PTG file instrument. The null hypothesis of the present study was that the tested irrigating solutions has similar effects on the cyclic fatigue resistance difference of ETP and PTG instruments.

## Methods

Sixty PTG and 60 ETP instruments were used in this study. All the instruments were F2 (25/0.08) and 25-mm long. All the instruments were inspected for defects and deformities at 13.6X magnification (Zeiss Pico; Carl Zeiss MediTec, Dublin, CA); no instrument was excluded. Each system was divided equally and randomly into three groups (n = 20 per group) based on the used irrigating solution; 17% EDTA, 5.25% NaOCl, or distilled water.

### Cyclic fatigue resistance experiment

**Fig. 1:**
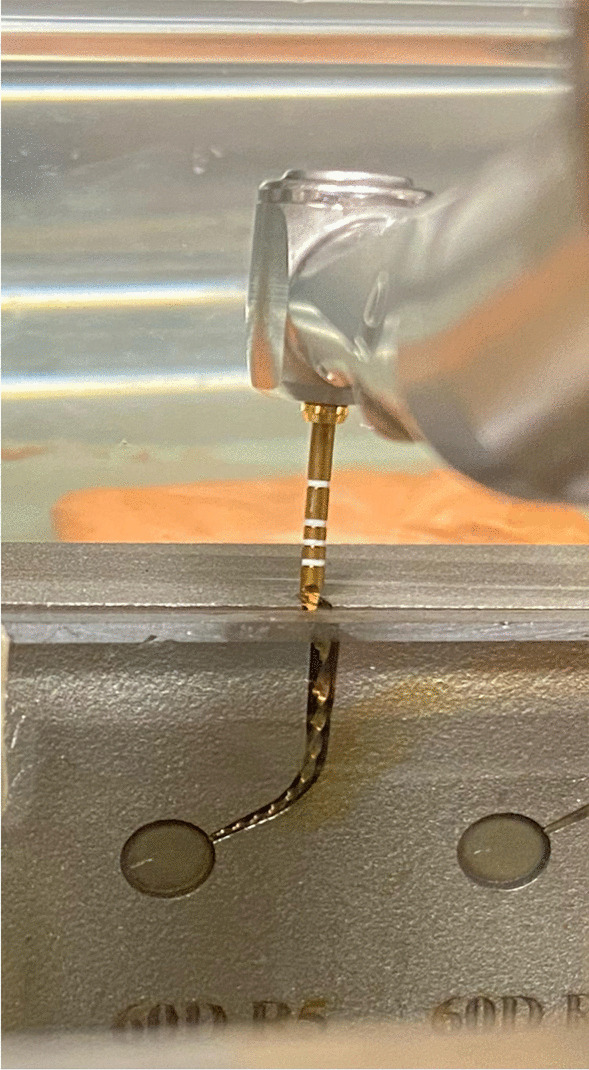
A custom-made stainless-steel canal with dimensions that are larger than the dimensions of F2 instrument by 0.1 mm in width. The canal has 60° curvature angle, 5 mm curvature radius and curvature center located 5 mm coronal to the instrument tip

Cyclic fatigue resistance was performed using artificial canals that were milled in stainless steel blocks using a laser micromachining technique (Fig. 1). The canals had a curvature angle of 60° and a curvature radius of 5 mm as described by Pruett et al. [25]. The center of maximum curvature was set at 5 mm from the instrument tip. Dimensions of the canals were larger than the dimensions of the F2 instrument by a 0.1-mm width [26]. A glass bar was used to cover the artificial canal to avoid instrument slippage and to visualize the instruments during rotation until it fractured. The block of artificial canals was stabilized inside a container that was filled with 17% EDTA, 5.25% NaOCl, or distilled water. The solution’s temperature was fixed at 37 with a tolerance limit of 1 °C as evident with a thermometer to simulate the body temperature. The endodontic rotary motor handpiece (TCM Endo III; Nouvag AG, Lake Constance, Switzerland) was mounted on a device that allowed reproducible and fixed positioning of the instrument. Each instrument was inserted inside the canal by 19 mm from its tip. The instrument was rotated at 300 revolutions per minute (rpm) until fracturing occurred. The time to fracture was recorded once the fracture was noticed audibly and visually by the aid of video recording. The number of cycles to fracture (NCF) was calculated using the following formula: NCF = rpm X time to fracture (in minutes). The artificial canal was replaced when any sign of corrosion was noticed. The fractured fragment's length was measured (in mm) using a digital stereomicroscope (Hirox-USA Inc, Hackensack, NJ).

### Scanning electron microscopy

From each group, three fractured instruments were randomly selected and cleaned with absolute alcohol in an ultrasonic bath for 3 min to remove debris on the surface. The fractured surfaces were observed under a scanning electron microscope (6360LV Scanning Electron Microscope; JEOL, Tokyo, Japan) to look for the topographic features of the fractured surface at a magnification of 180X.

### Statistical analysis

Since the data distribution was normal (Shapiro–Wilk test; *P* > 0.05), the data were analyzed statistically using one-way analysis of variance (ANOVA) and least significant difference (LSD) tests to compare different solutions within each experimental system. Within each solution, the experimental systems were compared using an independent t-test. The length of the fractured segment was analyzed using the Kruskal–Wallis test. Statistical analyses were conducted by using SPSS 20 software (IBM-SPSS Inc., Chicago, IL) at a confidence level of 95%.

## Results

**Fig. 2:**
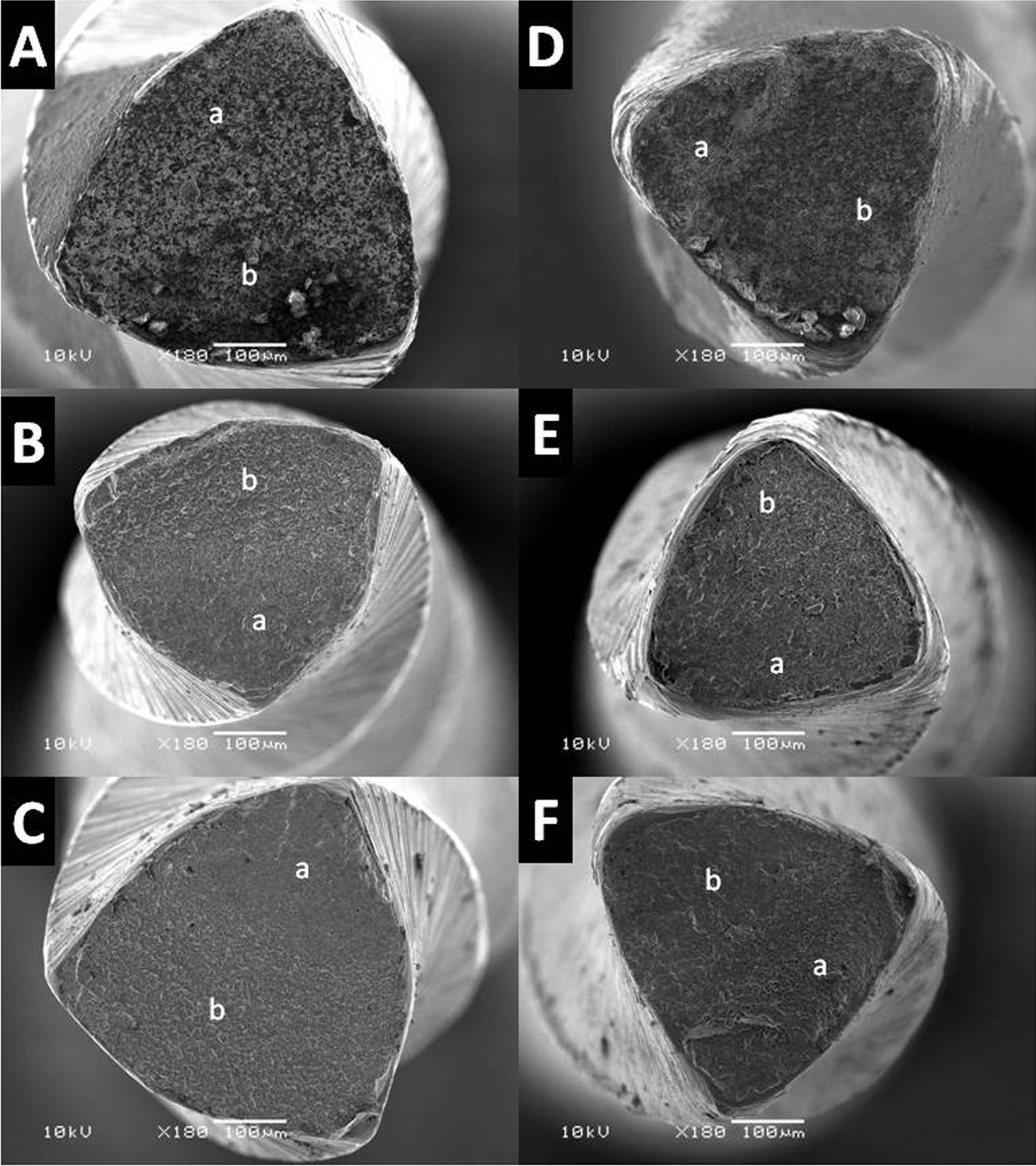
Scanning electron microscopic images of the ProTaper Gold (PTG) and EdgeTaper Platinum (ETP) instruments after cyclic fatigue tested in different solutions. **A** PTG in EDTA, **B** PTG in NaOCl, **C** PTG in distilled water, **D** EPT in EDTA, **E** EPT in NaOCl, and **F** EPT in distilled water. "a" corresponds to the fatigue striation area, and "b" corresponds to the fast fracture zone with dimples

ETP showed higher NCF than PTG in any of the tested solutions (*P* < 0.05). In each system, LSD tests showed that changing the solution from distilled water to EDTA did not affect the fatigue resistance (*P* > 0.05). However, NaOCl drastically decreased the NCF, compared to water and EDTA (*P* < 0.05) (Table 1). The scanning electron microscopy showed common features of cyclic fracture in the fractured surfaces of ETP and PTG instruments tested in different solutions, including the presence of fatigue striations and crack initiation area (Fig. 2). None of the tested surfaces showed any indication of corrosion in 17% EDTA, 5.25% NaOCl, or water (Fig. 2). The lengths of fractured fragments ranged from 4.15 to 4.60 mm (Kruskal–Wallis test; *P* > 0.05).

**Table 1:** Descriptive data (Mean ± SD) of cyclic fatigue resistance in the tested file systems

	Cyclic fatigue (Number of cycles to fracture)		
	EDTA	NaOCl	Distilled water
ProTaper Gold	979.0 ± 184.9^Aa^	659.4 ± 111.8^Ba^	939.4 ± 176.9^Aa^
EdgeTaper Platinum	1862.3 ± 365.7^Ab^	1422.8 ± 246.6^Bb^	1664.5 ± 285.9^Ab^

Different upper-case letters in a row indicate statistically significant differences between different solutions within each system (One-way analysis of variance (ANOVA) and least significant difference (LSD) tests; *P* ≤ 0.05). Different lower-case letters in a column indicate significant differences between 2 systems within each solution (independent t-test; *P* ≤ 0.05)

## Discussion

The current study examined the effect of EDTA and NaOCl on the cyclic fatigue resistance of ETP and PTG instruments when subjected to repetitive cyclic motions in an artificial canal at a temperature of 37 °C. In this study, ETP showed higher cyclic fatigue resistance than PTG in the tested solutions. The negative effect of NaOCl was evident in both groups; however, the fractographic appearance of ETP and PTG instruments cyclically fatigued in EDTA was similar to those fatigued in NaOCl and distilled water.

EDTA is used as a chelating agent and lubricant which comes in contact with the instrument during canal instrumentation [27]. This study showed that it has a negligible effect on the cyclic fatigue resistance of heat-treated instruments. Similarly, previous studies showed that EDTA did not have any effect on the cyclic fatigue resistance of other instruments [11, 14, 19]. However, another study reported that EDTA reduced the cyclic fatigue resistance of instruments studied after 3 min of immersion [13]. Moreover, this study also showed that NaOCl harmed the cyclic fatigue resistance of PTG and ETP instruments. The instrument was in brief contact with NaOCl; however, there was an apparent reduction in the NCF. This might be due to the developed corrosive zones, which could reduce the resistance to cyclic fatigue of the instrument [8, 12, 15, 18]. This is consistent with previous studies [10, 12]. However, other studies revealed that NaOCl did not reduce the cyclic fatigue resistance of other instruments [11, 13, 14, 19, 28]. These contradictory findings could be explained by differences in the implemented study methodology. The previous studies immersed the instruments in NaOCl or EDTA solution for different periods were then subjected to cyclic fatigue testing in canals filled with an oil lubricant. In our study, the instruments were not immersed before the test. Rather, they were subjected to cyclic fatigue by rotating inside an artificial canal which was filled with the tested solution at simulated body temperature. This would be more clinically relevant since it mimics the clinical condition. Besides that, the tested instruments in the abovementioned studies have different characteristics (alloy properties, instrument design, motion kinematics, and rotational speed) which might play a role in the performance of endodontic instruments in these tested solutions [11–15, 18, 19, 28, 29].

The ETP was introduced with a design similar to that of PTG and ProTaper Universal. The PTG was shown to have better resistance to cyclic fatigue and flexibility compared with the ProTaper Universal [20, 21]. However, it did not show improved resistance to torsional fatigue [30]. In this study, the ETP proved to have better cyclic fatigue resistance than PTG by almost 50% in any of the tested solutions. Consistently, a previous study done in water stated that ETP exhibited greater resistance to cyclic fatigue at simulated body temperature [24]. Although the tested systems are manufactured with heat treatment, have the same design and are used in continuous rotation at the same speed, their cyclic fatigue performance was different. This might be explained by differences in the thermo-mechanical properties of the systems as a result of manufacturers' variations and brand specification of heat-treated alloy. Further studies are required to investigate the metallurgical properties of ETP.

Clinically, the alternating use of NaOCl and EDTA is a popular regimen since it enhances the antimicrobial performance [31] and the removal of the smear layer [32]. However, this regimen might show a synergistic effect on the instrument's cyclic fatigue resistance. Conducting studies on NiTi instrument fracture in the presence of commonly used endodontic irrigating solutions would ensure a better imitation of clinical settings. This study used a high concentration of NaOCl which might be responsible for reducing the lifespan of the tested instruments [28]. Testing the instruments at lower concentrations might be more favorable for instrument performance. Therefore, further cyclic fatigue studies are needed to address this issue.

The utilized artificial canals were fabricated accurately using a laser micromachining technology to be similar to the tested instruments and to avoid differences in instrument positioning that might cause a discrepancy in the area of the fracture and consequently affect the cyclic fatigue measurement. It is worth mentioning that our results showed that the instruments fractured at the most curved areas with the range of 4.15–4.60 mm. This is consistent with previous results [10, 24] and confirmed the precise trajectory and positioning of the instruments during the test.

Changes in the transformation and metallurgical behavior are effective for exhibiting improvement in the performance of NiTi instruments [33]. Heat treatment is one of the approaches utilized to improve the fatigue resistance of instruments [33]. To reflect the actual clinical setting, the experimental temperature should be close to the intracanal temperature since it has been shown to have a major influence on the cyclic resistance behavior of conventional austenitic NiTi instruments as well as newly introduced NiTi instruments with special treatments [10, 34]. Moreover, a little decrease in the surrounding temperature or minimal stress application triggers the phase transformation in heat-treated instruments [20, 34]. For that, the current study tested the cyclic fatigue resistance of heat-treated instruments at simulated body temperature [35].

The tested F2 instruments have a variable taper changing over the length of its cutting blades. It shows 8% taper in the apical 3 mm of the instrument and regressively changes as we move upwards. This might not reflect the performance of other instruments with constant taper.

## Conclusions

Within the limitations of this laboratory study, ETP showed improved cyclic fatigue performance compared to PTG in all tested irrigating solutions. EDTA can be used in combination with NiTi instruments during canal instrumentation without compromising the cyclic fatigue resistance of PTG and ETP instruments. However, NaOCl drastically decreased the NCF of both systems.

## Data Availability

The datasets used and/or analyzed during the current study are available from the corresponding author on reasonable request.

## Abbreviations

PTG: ProTaper Gold
NiTi: Nickel–titanium
ETP: EdgeTaper Platinum
NCF: Number of cycles to fracture
NaOCl: Sodium hypochlorite
EDTA: Ethylenediaminetetraacetic acid
LSD: Least significant difference
